# Time series analysis of Sentinel 1 A SAR data to retrieve annual rice area maps and long-term dynamics of start of season

**DOI:** 10.1038/s41598-025-91655-z

**Published:** 2025-03-10

**Authors:** Pazhanivelan Sellaperumal, Ragunath Kaliaperumal, Muthumanickam Dhanaraju, Sudarmanian N.S, Shanmugapriya P., Satheesh S., Manikandan Singaram, Sivamurugan A.P, Raju Marimuthu, Baskaran Rangasamy, Tamilmounika R.

**Affiliations:** 1 Centre for Water and Geospatial Studies, Tamil Nadu Agricultural University, Coimbatore, 641003 India; 2 Department of Remote Sensing and GIS, Tamil Nadu Agricultural University, Coimbatore, 641003 India; 3 Tamil Nadu Rice Research Institute, Aduthurai, India

**Keywords:** Cropping patterns, Remote sensing, Rice area prediction, Sentinel 1A data, Plant sciences, Environmental sciences

## Abstract

Rice is a vital staple crop globally, and accurate estimation of rice area was crucial for effective agricultural management and food security. Synthetic Aperture Radar (SAR) data has emerged as a valuable remote sensing tool for rice area estimation due to its ability to penetrate cloud cover and capture backscattered signals from rice fields. The backscatter signature of rice showed a minimum dB value at agronomic flooding indicating the Start of Season (SoS). The parameters viz., the minimum values of −22.03 to −17.69 dB at the start of season, maximum value of −16.10 to −14.20 dB at the peak of season coinciding with heading and corresponding mean increase of 5.07 dB during growing stages were utilized for developing rule-based classification system. Rice area was estimated over the Cauvery Delta Zone of Tamil Nadu, India for the past six years during *samba* (August–January) season from 2017 to 2023 using Sentinel 1 A Synthetic Aperture Radar satellite data. Rice area maps were generated for the region utilizing parameterization with a classification accuracy of 88.5 to 94.5 per cent with a kappa score of 0.77 to 0.87 during the study period. The total classified rice area during *samba* season in the Cauvery Delta Zone was 508,581 ha, 456,601 ha, 506,844 ha, 511,714 ha, 524,723 ha and 476,586 ha for the years 2017–18 to 2022–23, respectively. The Start of Season (SoS) maps for *samba* season revealed that the major planting periods for rice were between the second fortnight of September to first fortnight of November in all the years except 2018 when early planting happened during the first fortnight of September due to favorable weather conditions and assured water supply. Near real-time information on rice area, start of season, and progress of planting derived using SAR satellite data will facilitate the development of decision support systems for sustaining the productivity of rice-based ecosystems.

**Supplementary Information:**

The online version contains supplementary material available at 10.1038/s41598-025-91655-z.

## Introduction

Rice is an important staple crop in many South and Southeast Asian nations, as around 60 per centof the world population consumes rice. India is the second-largest producer of rice worldwide, with 47 million hectares of area under rice cultivation, which is 27.6 per cent of the total cultivated area, with an annual production of 132 million metric tons of rice. India contributes 20 per cent of the world’s rice production, making it important to assess the crop area in time with more precision. According to the FAO^1^, India’s average consumption per capita/year was ~ 68.2 kg of rice, and the global annual per capita rice consumption is more than 50 kg. High population growth with changing consumer preferences has caused rapid expansion in rice consumption, and the production was estimated to be 555 million tons in 2035^2^. Accurate and consistent information on the area under production was essential for national and state-level planning. This information plays vital role in the policy decisions related to imports, exports, and prices directly influencing food security. The traditional crop area estimation method, which involves a vast labor force, was tedious, time-consuming, erroneous, and practically impossible to implement at a large scale. The agricultural policy program currently depends on the timely gathering of information *via* field and aerial surveys. Operational systems provide accurate data but have several inherent drawbacks, including difficulties in comparing statistics and validating information collected by various agencies. They currently use different methodologies for monitoring agricultural production, but production estimates are available close to harvest time, time-consuming and expensive due to frequent field trips and surveys.

Remote sensing technology has been used in agriculture for several decades and the recent developments in this field have made it more accessible to farmers and ranchers. Remote sensing is the use of satellite images that take photos of a field over time so that the grower can analyse conditions based on the data and take action that will have a positive influence on crop growth. Satellite imaging and machine learning, which can make precise forecasts about rice area and productivity, are presently used in a variety of ways. The current agricultural policy program relies on timely information collected through field and aerial surveys. Although operational systems deliver accurate data, they come with a variety of intrinsic flaws, such as challenges in comparing statistics and authenticating data gathered by different agencies. Different approaches are currently being used to monitor agricultural production, but estimates are only available just before harvest and also time/money-consuming due to the frequent fieldwork and surveys.

Optical remote sensing data is primarily impacted by cloud cover. Since rice crops mainly grow during the rainy season, cloud cover presents a significant challenge, making optical data essential for accurate crop area mapping. Since 1998, operational rice acreage estimation in India has been done using synthetic aperture radar (SAR) data^3^. Recent developments in Synthetic Aperture Radar (SAR) sensors have made it possible to calculate rice acreage, seasonality and days of floods. Multi-temporal SAR data can be used to estimate rice area during different stages of the growing season, which can improve accuracy^4^. Supervised crop classification from Sentinel-1 SAR data VV, VH and VV/VH time series to classify crops with an overall accuracy of more than 70 per cent. Integrating the multi-temporal optical and SAR data using the GEE machine learning algorithm for mapping the rice fields^5^. Cloud cover can be an issue for mapping and monitoring the state of the rice crop, but recent and upcoming Synthetic Aperture Radar (SAR) sensor deployments, together with cutting-edge automated processing, can offer long-term answers^6^.

For assessing crop area/loss at the farm or village level, high-resolution SAR data can be employed, which can produce more precise estimations^7^. Studies have shown that rice area maps generated from SAR data can have high precision, ranging from 90.7 to 94.7 per cent^7–9^. Mapping and monitoring of rice-growing areas in Tamil Nadu using COSMO SkyMed and TerraSAR-X datasets SAR imageries of high resolution and determined cropping extent and rice growth. CSK data are available from four X-band HH-SAR satellites with a 3.12 cm wavelength and a 16 day revisit period for the same satellite with the same observation angle. TSX is provided by one X-band HH SAR satellite with a 3.11 cm wavelength and 11 day revisit period with the same observation angle at strip map mode (3 m resolution) with a footprint of 30 × 50 km and Scan SAR mode (10 m resolution) with a footprint of 100 × 150 km^10^. With the latest addition, Sentinel 1 A and 1B data are available from the European Space Agency (ESA) at C band with a spatial resolution of 5 m and 20 m with a temporal resolution of 12 days individually and 6 days in combination.

A rule-based classification approach and parameter selection approach are available for rice mapping in which the rules and parameters are derived from agronomic knowledge of the rice crop and its management^11^. Rice area maps and Start of Season maps resulted in 87 to 90 per cent accuracy through rule-based classification. Monitoring the small rice fields of Southern China using TerraSAR-X data and achieved an accuracy of 90 per cent^12^. Lowland rice can easily be found with SAR imaging, especially in tropical areas with constant cloud cover. A new era for SAR-based agricultural monitoring is beginning with the launch of the C-band Sentinel-1 mission, the X-band TerraSAR-X mission, the commercial-grade Capella satellites and the impending NASA-ISRO Synthetic Aperture Radar (NISAR) program scheduled for the coming years. The main objective of this study is to evaluate the long-term capability of SAR systems to delineate rice crop areas every 12 days using parameterized classification.

## Methodology

### Study area

**Fig. 1:**
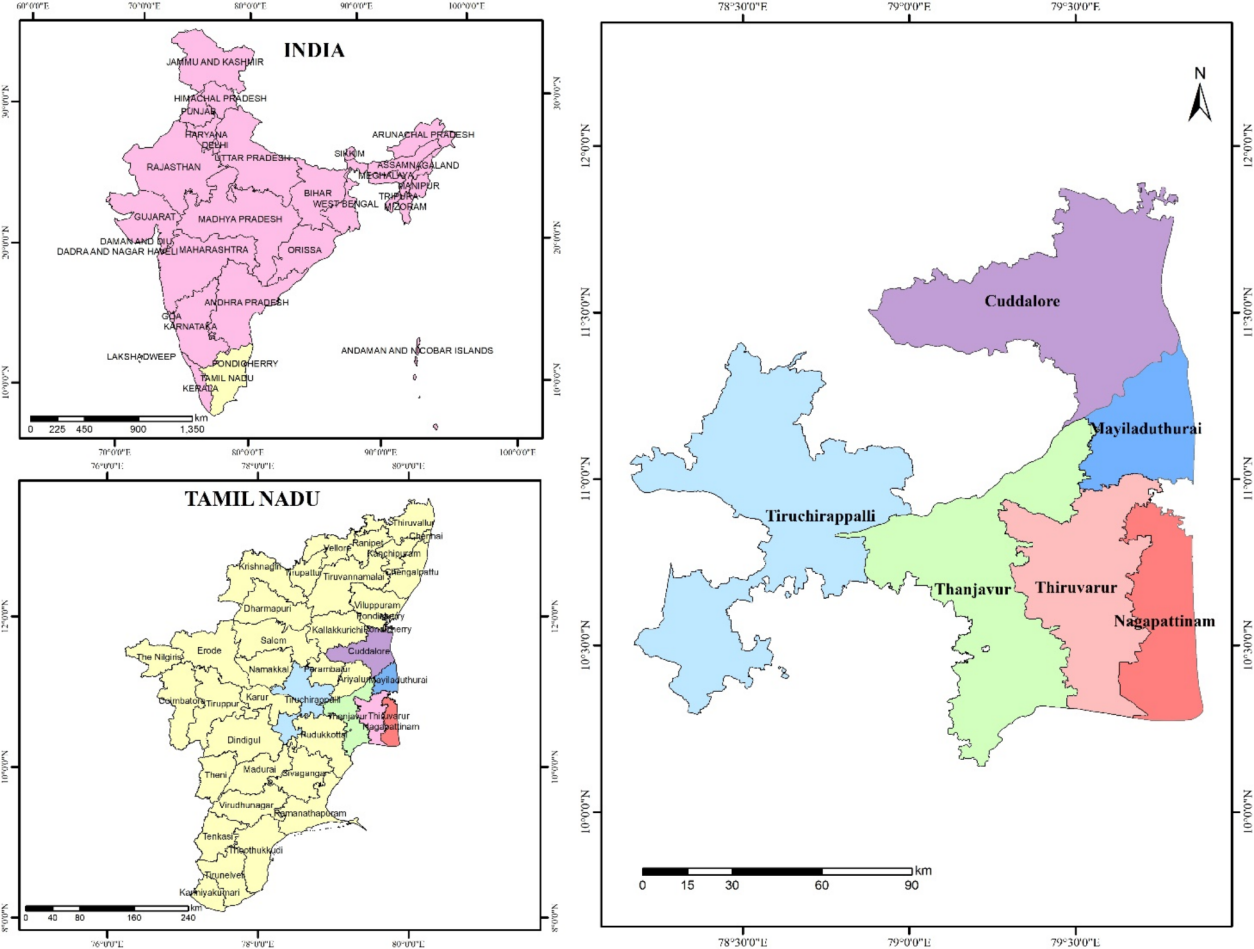
Study area in Cauvery Delta Zone of Tamil Nadu.

Cauvery Delta Zone lies in the eastern part of Tamil Nadu comprises of Cuddalore, Nagapattinam, Thanjavur, Thiruvarur and Tiruchirappalli districts (Fig. 1). It is geographically bounded by Cuddalore district in the North, Bay of Bengal in the East, the Palk straight in the South and Tiruchirappalli district in the West. The study area geographically lies between 78° 15’ to 79° 45’ East longitudes and 10°00’ to 11°30’ North latitudes with an altitude of 90 m. In March 2020, the Nagapattinam district was bifurcated into two districts, namely, Nagapattinam and Mayiladuthurai. For analysis purposes, the districts were combined and used in this study.

**Fig. 2:**
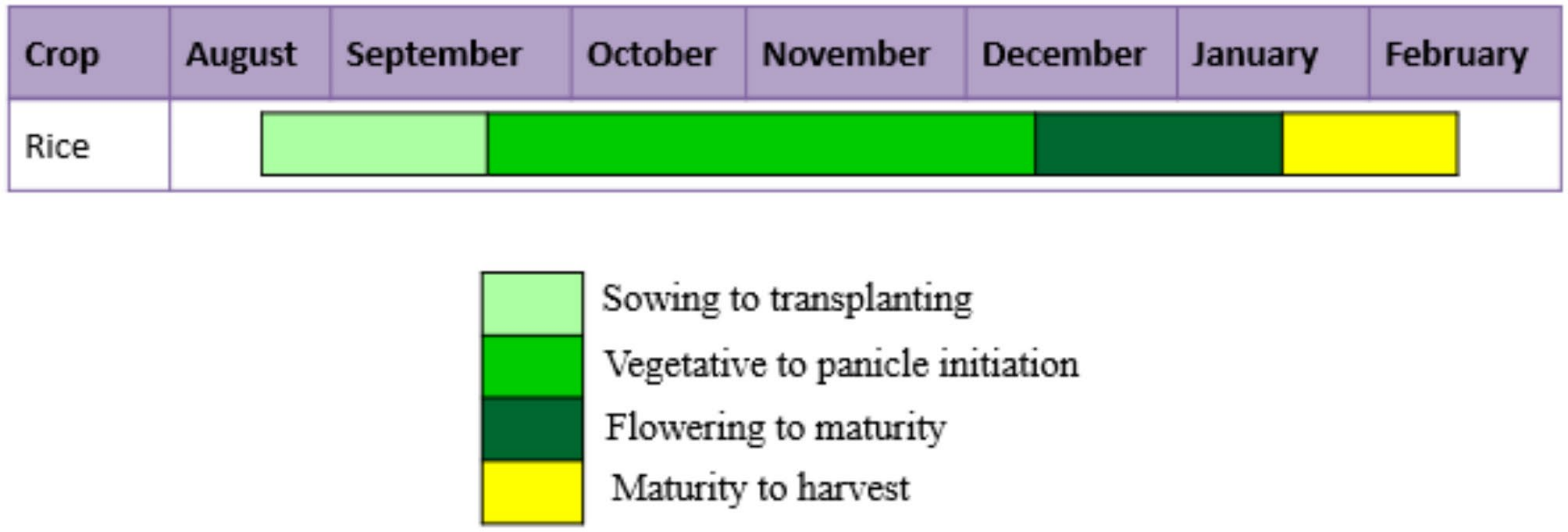
Rice crop calendar of the study area.

Soil types of different districts of Cauvery Delta Zone are as follows, the soils of Tiruchirapalli are Alluvial sandy loam and loamy soil. Thanjavur district has alluvial soil in Cauvery delta and sandy soils in coastal area. The predominant soil types in Thiruvarur district are sandy, coastal alluvium and red loam. In Nagapattinam, sandy coastal alluvium is the predominant soil type. The major crops grown in the district are rice, pulses (Blackgram and Greengram), banana, sugarcane, cotton, sorghum, groundnut and gingelly. Rice is the most extensively cultivated crop in this zone with three seasons, namely *Kuruvai (*June-August), *Samba* (August-January) and *Thaladi* (January-March). This zone is also known as the ‘rice bowl’ of Tamil Nadu with Rice as the principal crop is grown either as a single or double-crop. The rice crop calendar of the study area is given in Fig. 2.

### Sentinel 1A - synthetic aperture radar (SAR) data

Synthetic Aperture Radar (SAR) can acquire data during day or night under all weather conditions with the advantage of overcoming cloud cover. Furthermore, Sentinel 1 A, with its C-SAR instrument, can offer reliable, repeated wide-area monitoring (Table 1). Sentinel 1 A is a SAR sensor that operates in the microwave region launched by the European Space Agency in 2014. It provides dual polarization of VV (Vertical–Vertical) and VH (Vertical–Horizontal) polarization, and it has a temporal resolution of 12 days and a spatial resolution of 20 m.

**Fig. 3:**
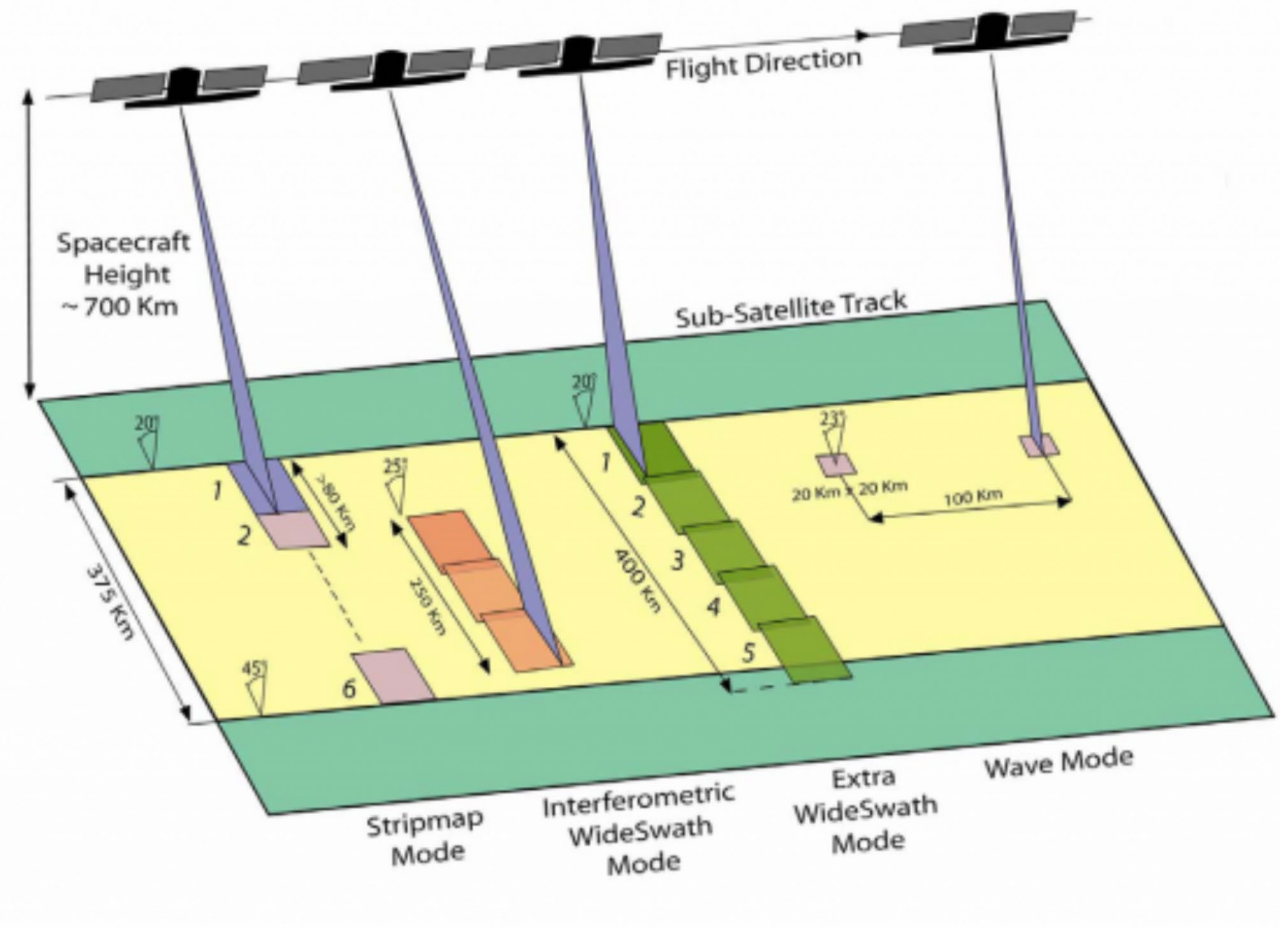
Sentinel 1 A product modes.

Sentinel 1 A has four standard operational modes, designed for inter-operability with other systems (Fig. 3) The Sentinel 1 A Level-1 Ground Range (GRD) Products were downloaded during crop growing period from August to February at 12 days intervals from https://scihub.copernicus.eu/dhus/. The overview of the Sentinel 1 A data acquisitions and coverage over the study area is presented in Table 2.

**Table 1:** Details of Sentinel 1 A (IW-GRD) data^13^.

Parameters	Characteristics
Pixel value	Magnitude detected
Coordinate system	Ground range
Polarizations	Single (VV), Cross (VH)
Ground range coverage (km)	251.8
Radiometric resolution (dB)	1.7
Bits per pixel	16
Resolution (range x azimuth) (m)	20.4 × 22.5
Pixel spacing (range x azimuth) (m)	10 × 10
Incident angle	32.9^o^
Number of looks	5 × 1
Range look bandwidth (MHz)	14.1
Azimuth look bandwidth (Hz)	315
Equivalent number of looks (ENL)	4.4
Absolute location accuracy (m) (NRT)	7

**Table 2:** Data acquisition schedule of Sentinel 1 A.

Year	Data acquisition period	No. of acquisitions
2017–18	9th August, 2017 to 12th January, 2018	14
2018–19	16th August, 2018 to 19th January, 2019	13
2019–20	11th August 2019 to 7th February 2020	15
2020–21	5th August, 2020 to 25th February, 2021	16
2021–22	12th August, 2021 to 3rd January, 2022	13
2022–23	19th August, 2022 to 10th January, 2023	12

### Software used for analysis

High-resolution Sentinel-1 A (SAR data) involves rigorous pre-processing, which is time-consuming and tedious. Hence, this study utilizes specialized software customized to perform the sequential steps of SAR data processing and analysis with advanced GIS tools. The data processing and spatial analysis were achieved with the following software packages:

- MAPscape: SAR data processing and rice area estimation.
- ArcGIS and QGIS: Handling spatial data sets and GIS operations like mapping from optical and SAR data.
- MS-Excel: Generation of graphs and statistics deriving.

### Ground truth collection

**Fig. 4:**
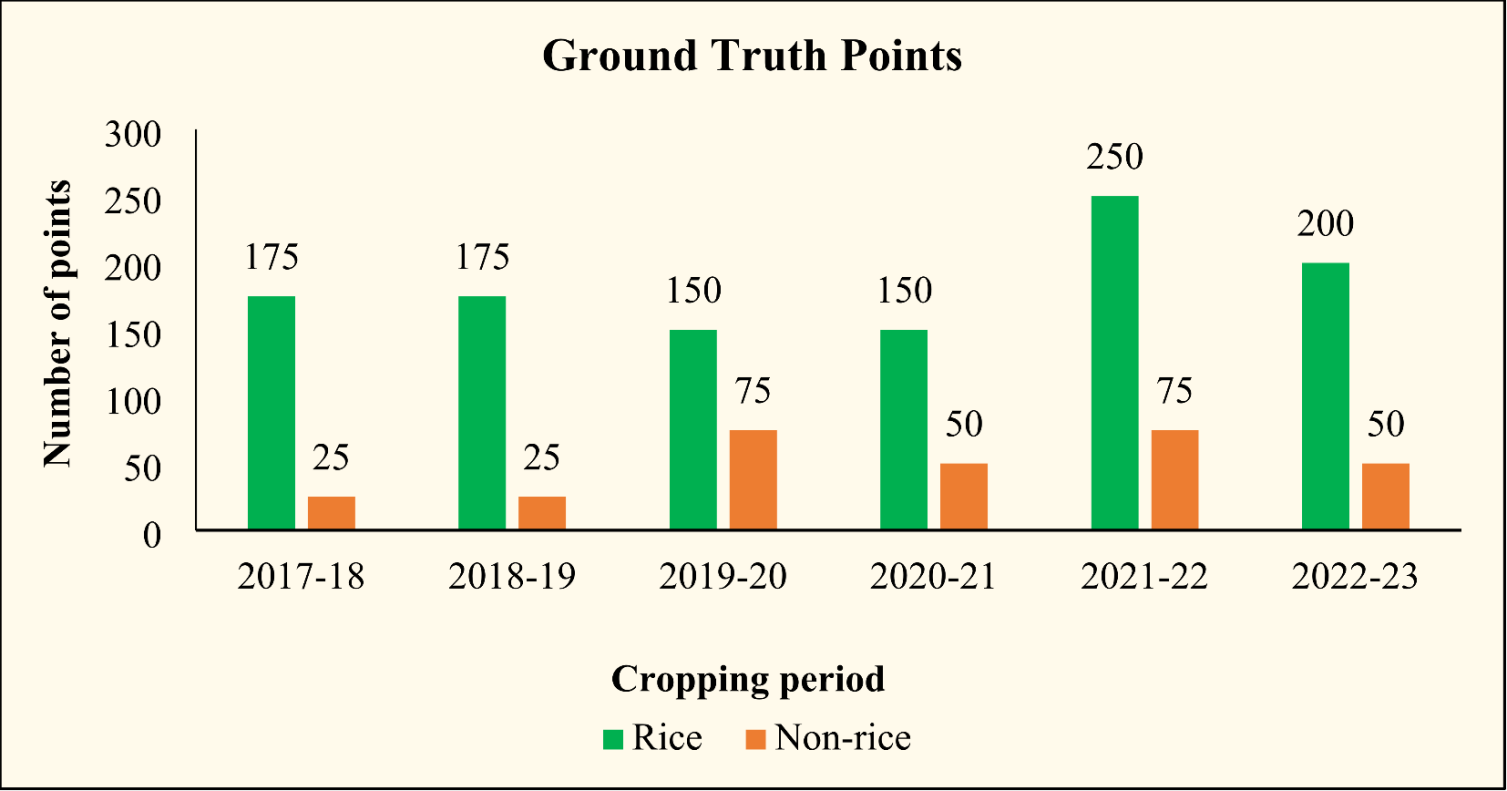
Rice and non-rice points collected over the years.

Ground truth surveys are conducted throughout the study area from 2017 to 2023 for s*amba* season to assemble land cover information to validate rice estimates derived from satellite data. Observations are taken during the image acquisition date, which include latitude and longitude from handheld GPS receivers, descriptions of the area and object, photos of the field’s status, plant height, and crop stage. The rice and non-rice points collected during the study period are given in below Fig. 4. The significant role of ground truth points for crop discrimination and acreage estimation was emphasized^14–16^. 60 per cent of the ground truth points collected were used for training the classification process, while the remaining 40 per cent of the data were used for validation^17^.

### Basic processing of SAR data for multi-temporal analysis

**Fig. 5:**
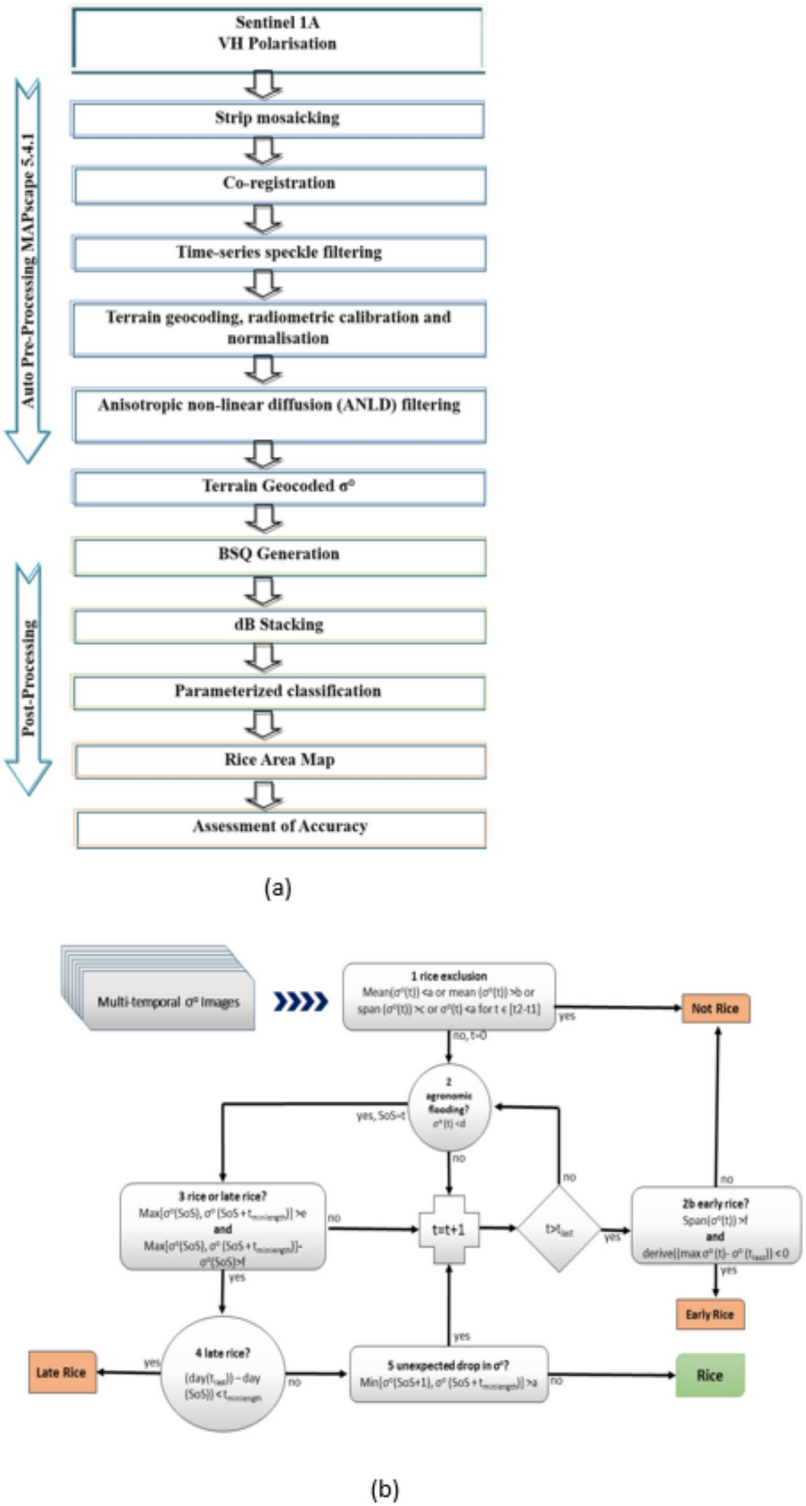
(**a**) Flow chart depicting Sentinel 1 A satellite data processing for rice area estimation. (**b**) Rule-based rice detection algorithm for multi-temporal C-band σ° in MAPscape-RIICE Software.

MAPsacpe-RIICE, a customized software developed by Sarmap, Switzerland was used for basic processing of time series Sentinel 1 A SAR data from ESA. A fully automated SAR processing chain composed was incorporated in the software module to transform sentinel 1 A IW – GRD multi-temporal SAR data into terrain geo-coded σ° values^18^. The basic processing was executed sequentially as detailed below and given in Fig. 5a.

#### Strip mosaicking

Mosaicking of single frame SAR datasets of the same orbit and acquisition date.

#### Co-registration

As a prerequisite for time series speckle filtering, multi-temporal images acquired in the same observation geometry were coregistered using this tool.

#### Time-series speckle filtering

As proposed an optimum multi-temporal filter was applied to balance differences in reflectivity between images at different times^19^.

#### Terrain geocoding, radiometric calibration and normalization

The Digital Elevation Model (DEM) was used to transform positions of σ° elements into slant range image coordinates. During the process, three-dimensional object coordinates in a cartographic reference system were converted into two-dimensional coordinates of slant range images using the range-Doppler approach. Radiometric calibration was performed using the radar equation, which considers scattering area, antenna gain patterns and range spread loss. To compensate for the range dependency, σ° was normalized according to the cosine law of the incidence angle.

#### ANLD filtering

Homogeneous targets were smoothened using ANLD filter by enhancing the difference between neighboring areas^20^.

#### Removal of atmospheric attenuation

The thick layer of water vapor present in the atmosphere and heavy rainfall affects the penetrating microwaves during SAR-based remote sensing due to which the values of backscattering co-efficient decreased or increased. An interpolator is used to identify and process the temporal signature anomalies in terms of peaks and troughs and rectify the errors^21^.

#### Multi-temporal Σ° rule-based rice detection

The multi-temporal stack of terrain-geocoded σ° images was analyzed through a rule-based rice detection algorithm in MAPscape-RIICE. The temporal evolution of σ° was studied from an agronomic perspective, which also required a priori knowledge of rice maturity, calendar and duration and crop practices from field information and knowledge of the study location. The rule-based rice detection methodology (Fig. 5b) uses parameters derived from temporal signature generated with VH polarization. Hence, dB curves for rice fields were constructed by extracting temporal signatures in VH polarization for each monitoring field. The temporal signatures developed from the selected sites for paddy crop were used to retrieve the parameters viz., lowest mean, highest mean, maximum variation, max value at SoS, min value at the peak, minimum variation, etc., as input for the classification. The quality of area extraction depends on the parameters used in delineating paddy pixels for SAR satellite data. The efficiency of parameterized classification in delineating rice crops was demonstrated in studies conducted^22,23^ for rice crops in increasing the accuracy levels.

**Table:** 

a = Lowest mean b = Highest mean c = Maximum variation d = Maximum value at SoS t_2_-t_1_ = Maximum time underwater	e = Minimum value at maximum peak f = Minimum variation t = Time SoS = Start of season
t_minlength_ = Minimum number of days of season length t_maxlength_ = Maximum number of days of season length t_last_ = Date of the last acquisition	

### Accuracy assessment

The accuracy assessment is a comparison of the rice area map against ground truth data. Validation points are split into two classes namely, rice and non-rice points. A standard confusion matrix was applied to the rice/non-rice validation points collected at each site. The accuracy of the rice area map was assessed through the confusion matrix using the ground truth points to classify rice and non-rice pixels^24^.

The Error matrix and Kappa statistics are used for evaluating the accuracy of the estimated rice area. The class allocation of each pixel in the classified image is compared with the corresponding class allocation on reference data (Crop Cutting Experiment data) to determine the classification accuracy. The pixels of agreement and disagreement are compiled in the form of an error matrix. The rows and columns represent the number of all classes, and the elements of the matrix represent the number of pixels in the testing dataset^25^.

The accuracy measures, such as overall accuracy, producer’s accuracy and user’s accuracy, are estimated from the error matrix^26^. The overall accuracy, which is the percentages of correctly classified cases lying along the diagonal, was determined as follows:

$$\:\begin{array}{cc}\text{Overall\:Accuracy\:=}&\:\frac{\sum\:\left(\text{Correctly}\text{}\text{classified}\text{}\text{classes}\text{}\text{along}\text{}\text{diagonal}\right)}{\sum\:\left(\text{\:Row\:Total}\text{}\text{or\:Column}\text{}\text{Total}\right)}\end{array}$$

The producer’s accuracy (errors of omission) of each class was computed by dividing the number of samples that were classified correctly by the total number of reference samples as follows:

$$ \begin{array}{*{20}c}    {\Pr oducer's~Accuracy~~ = } & {\frac{{Number~of~correctly~classified~classes~in~a~column}}{{~Total~number~of~items~verified~in~that~column}}}  \\   \end{array}  $$

The user’s accuracy (errors of commission) of each class was computed by dividing the number of correctly classified samples of that class by the total number of samples that were verified as belonging to the class as follows:

$$ User's\,Accuracy\, = \,\frac{{Number\,of\,correctly\,classified\,items\,in\,a\,row}}{{Total\,number\,of\,items\,verified\,in\,that\,row}} $$

#### Kappa coefficient

Another measure of classification accuracy is the kappa coefficient, which measures the proportional (or percentage) improvement by the classifier over a purely random assignment to classes^27^. The kappa coefficient can be estimated from the formula given below.

$$\:\widehat{K}=\frac{\text{N}\text{A}-\text{B}}{{N}^{2}-B}$$

For an error matrix with r rows, and hence the same number of columns, Where,

A = the sum of r diagonal elements, which is the numerator in the computation of overall accuracy.

B = sum of the r products (row total x column total).

N = the number of pixels in the error matrix (the sum of all r individual cell values).

## Result and discussion

### Rice area estimation from 2017 to 2023

**Fig. 6:**
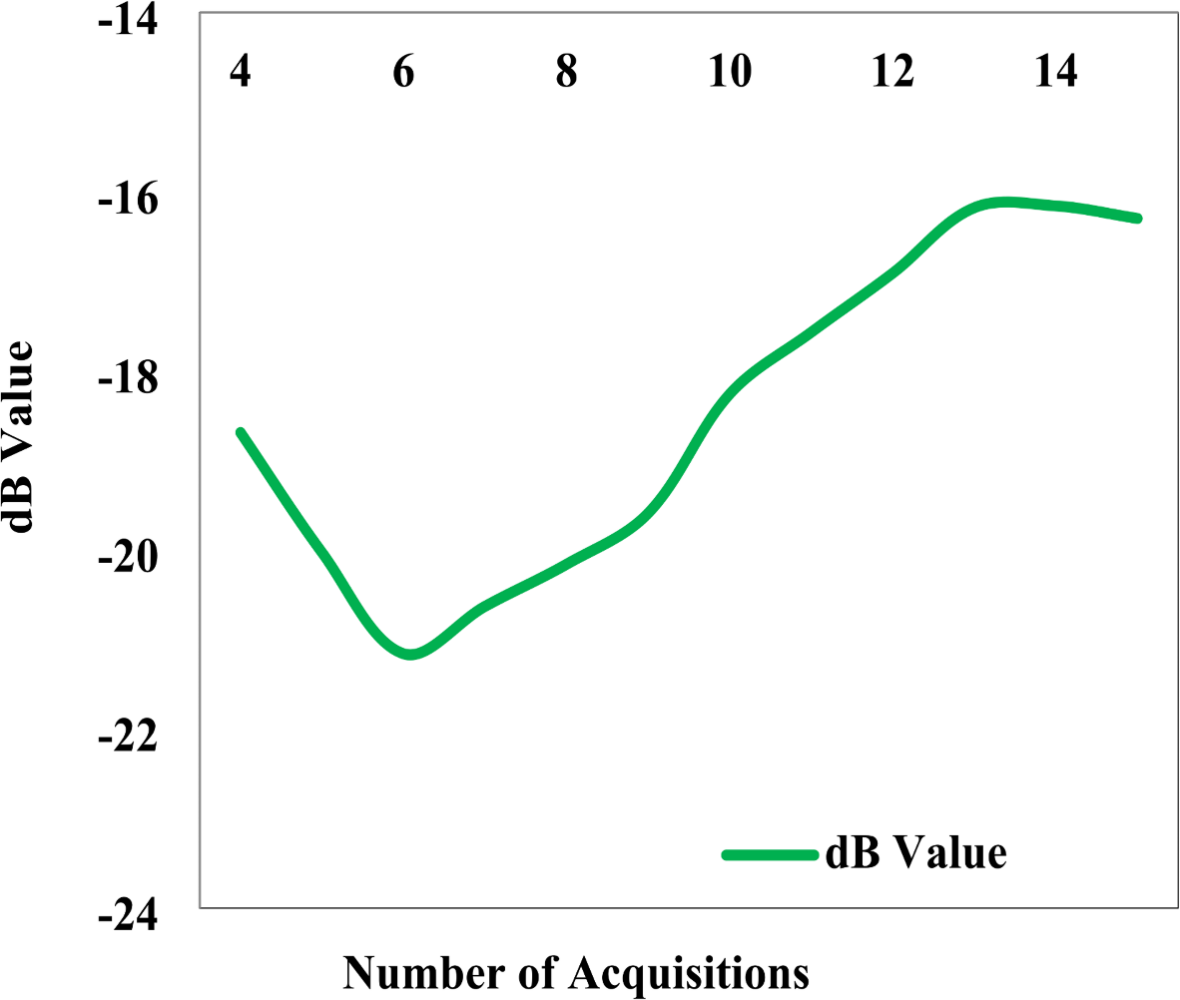
dB curve for rice.

**Fig. 7:**
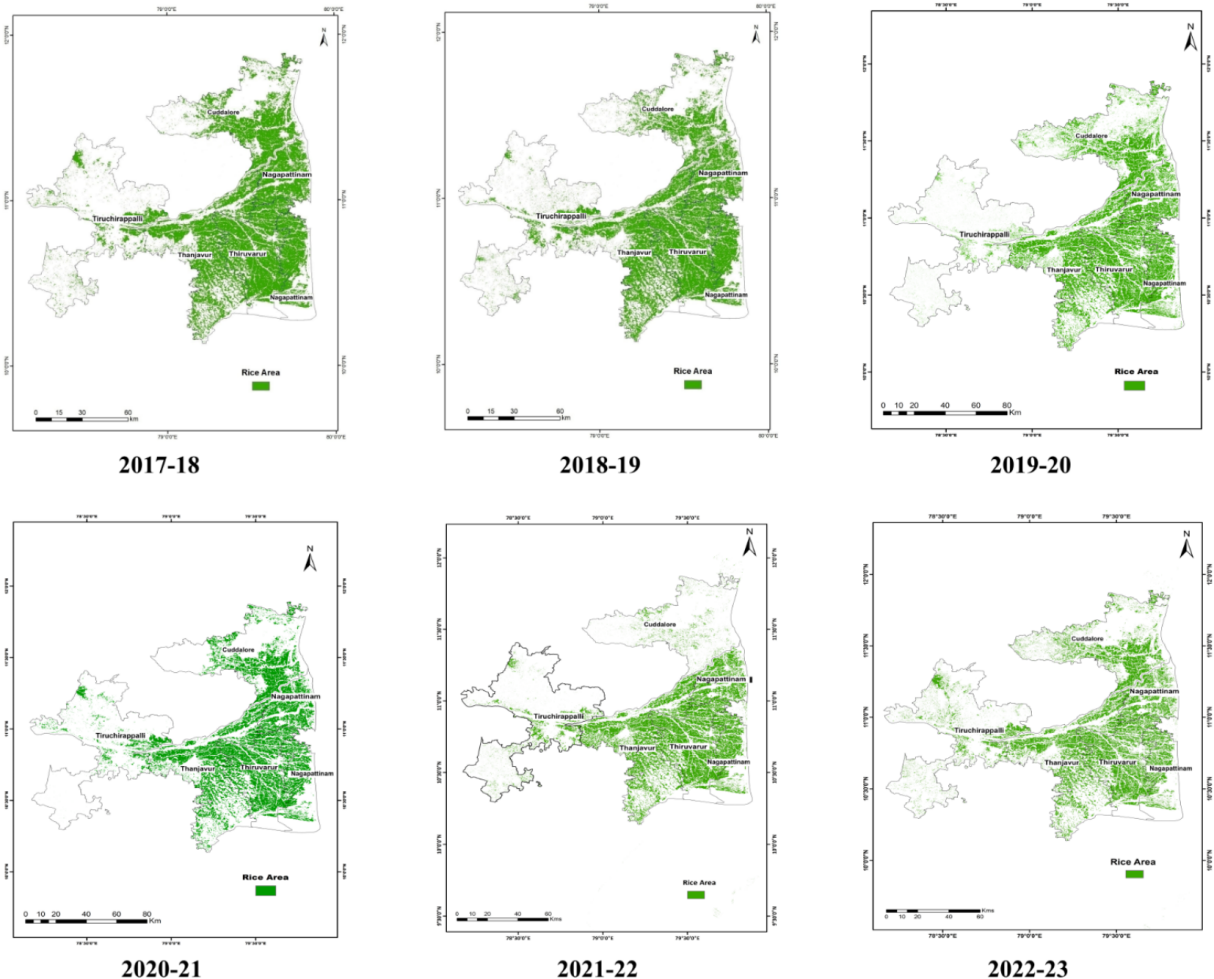
Rice area map of the Cauvery Delta Region during 2017 to 2023.

Sentinel 1 A SAR satellite datasets were acquired at 12 day intervals with 20 m spatial resolution during the crop growing season of study area. Sentinel-1 A has the ability to delineate single and double cropping in rice through VH backscatter patterns^28^. The C-band datasets (Sentinel-1 and RADARSAT-2) were found to be more effective at the early stages of crop and more sensitive to biomass, meanwhile L-band dataset (UAVSAR) was more effective at the later stages^29^. The delineation of rice pixels from SAR data depends on the parameters derived from the temporal backscatter values during the crop growth period^30^. The backscattering coefficient (Fig. 6) and multi-temporal features were extracted and used to map the rice area. Rice area statistics and maps are given in Table 3; Fig. 7.

Generally, the backscatter signature of rice showed a minimum dB value at agronomic flooding, a peak at maximum tillering to flowering stage, and it declines after that. The minimum values at start of the season of rice ranged from − 22.03 to -17.69 dB. The maximum values corresponding to the flowering stage ranged from − 16.10 to −14.20 dB. The increase in dB corresponding to crop growth from seedling to flowering stage ranged from 2.69 to 6.74 dB with a mean value of 5.07 dB. Crop density and height of rice increases backscatter coefficient, drying of plant decreases the backscatter coefficient. VV backscatter was higher than VH due to the attenuation from paddy stems^31^. Many research was carried out to understand the relationship between backscatter and crop growth and apply them to identify and monitor crop growth^9,21,30^.

During 2017-18, in the Cauvery Delta Zone, a total of 508,581 ha of rice area was delineated from the multi-temporal Sentinel 1 A SAR data using a parameterized classification integrating multi-temporal features. Among the districts, Thiruvarur recorded the highest area of 132,258 ha followed by Thanjavur and Nagapattinam with an area of 126,226 and 119,411 ha, respectively. Cuddalore and Tiruchirappalli districts registered an area of 99,170 and 31,516 ha, respectively. In 2018-19, the rice area was assessed using remote sensing data and there was a reduction in rice area was observed with a value of 456,601 ha. Among the districts, Thiruvarur recorded the highest area of 126,019 ha followed by Thanjavur and Nagapattinam with an area of 124,618 and 105,107 ha, respectively. Cuddalore and Tiruchirappalli registered an area of 77,312 and 23,545 ha, respectively.

A total of 506,844 ha rice area was delineated during *samba* season in 2019-20. Among the districts, Thanjavur recorded the highest area of about 141,287 ha, followed by Thiruvarur and Nagapattinam with an area of 125,589 ha and 117,761 ha, respectively. Cuddalore and Tiruchirapalli accounted for the rice area of 104,331 ha and 17,877 ha, respectively. A total of 511,714 ha of rice area was delineated during *samba* season 2020-21. Among the districts, Thanjavur recorded the highest area of about 141,077 ha, followed by Thiruvarur and Nagapattinam with an area of 127,752 ha and 110,938 ha, respectively. Other districts Cuddalore and Tiruchirapalli registered rice areas of 88,002 ha and 43,944 ha, respectively.

In 2021-22, a total of 524,723 ha area was under rice cultivation with which Thanjavur recorded the highest rice area of 139,171 ha, while Tiruchirapalli recorded the least rice area of 32,484 ha. Thiruvarur, Cuddalore and Nagapattinam accounted for rice area of 127,028 ha, 101,821 ha and 124,219 ha, respectively. During 2022-23, a similar trend was observed. However, there was a reduction in rice area as well as district-wise distribution. The change is attributed to the reason that due to complementary weather conditions and water storage, part of the single-cropped rice area was converted to double-cropped areas^16,32^.

**Table 3:** Rice area (ha) for the cauvery Delta districts from 2017 to 2023.

Sl. no.	Districts	2017–18	2018–19	2019–20	2020–21	2021–22	2022–23
1	Thanjavur	126,226	124,618	141,287	141,077	139,171	117,907
2	Thiruvarur	132,258	126,019	125,589	127,752	127,028	110,512
3	Nagapattinam	119,411	105,107	117,761	110,938	124,219	102,792
4	Cuddalore	99,170	77,312	104,331	88,002	101,821	100,348
5	Tiruchirapalli	31,516	23,545	17,877	43,944	32,484	45,027
Total		508,581	456,601	506,844	511,714	524,723	476,586

### Confusion matrix for accuracy assessment of SAR-based rice area estimation

A confusion matrix was formed to assess the accuracy of rice area maps by conducting ground truth collection on a rice/non-rice basis, where all land types other than rice classes were classified as non-rice classes given in Table 4.

**Table 4:** Confusion matrix for accuracy assessment of SAR-based rice area estimation.

Year	Rice	Non-rice	Overall accuracy (%)	Kappa index
2017–18	175	25	88.5	0.77
2018–19	175	25	91.5	0.83
2019–20	150	75	89.3	0.79
2020–21	150	50	94.5	0.89
2021–22	250	75	91.7	0.83
2022–23	200	50	93.3	0.87

In total, 200 validation points covering 175 rice and 25 non-rice points were collected during 2017-18 and used for validation of the rice area map of the Cauvery Delta Zone. Considering the efficiency of the methodology of mapping rice areas with SAR data, the overall accuracy was 88.5 per cent. The Kappa Coefficient was 0.77 indicating good accuracy levels of the products. The phenology-based classification approach from Sentinel-1 A SAR data, in general, has provided an accuracy of more than 70 per cent which makes the data dependable for crop monitoring^23^. During 2018-19, a total of 200 ground truth points were collected covering 175 rice and 25 non-rice points and used in validating the rice area map. Cauvery Delta Zone ground truth points were classified with an overall accuracy was 91.5 per cent and the Kappa Coefficient was 0.83.

In total, 225 ground truth points have been collected in the study area during 2019-20 consisting of 150 rice and 75 non-rice points. Points were classified with an overall accuracy of 89.3 per cent and Kappa Coefficient of 0.79. In 2020-21, a total of 200 ground truth points were collected randomly covering 150 rice and 50 non-rice points during the crop growing season in the study area. The overall accuracy of the rice area map was 94.5 per cent with a Kappa Coefficient of 0.89. Around 325 ground truth points were collected during the field survey comprising 250 rice points and 75 non-rice points in 2021-22. As a result, the overall accuracy of the rice area map was 91.7 per cent, and the kappa coefficient was 0.83. In 2022-23, around 250 ground truth points were collected comprising 200 rice points and 50 non-rice points. The overall accuracy of the rice area map was 93.3 per cent, and the kappa coefficient was 0.87.

### Start of the season (SoS)

**Fig. 8:**
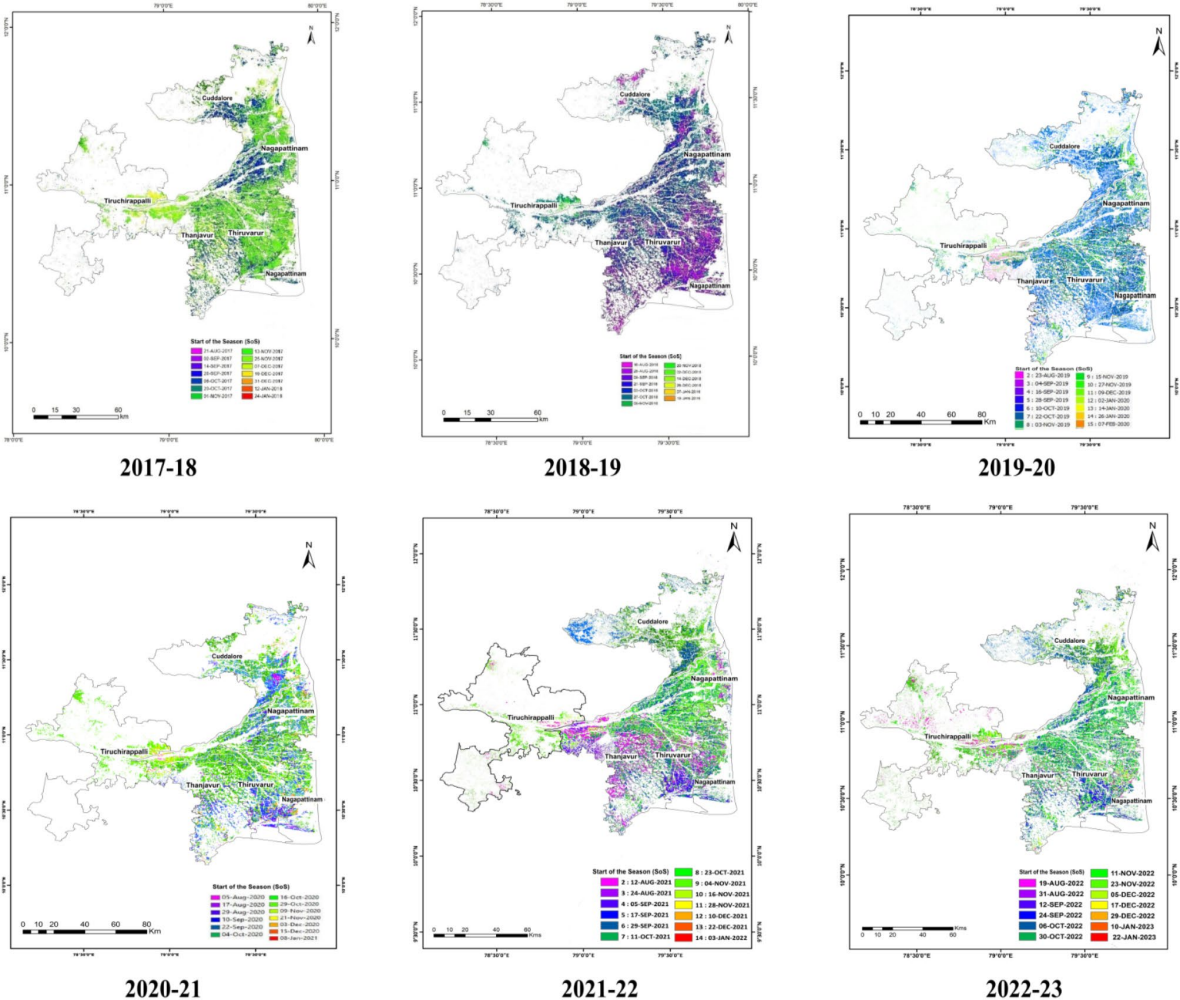
District wise rice area (ha) at 12 day intervals from start of the season maps in Cauvery Delta Zone using Sentinel-1 A.

Start of the season maps and statistics were generated for the rice area using the threshold of minimum dB in the backscattering for each pixel from the Sentinel 1 A SAR data and given in Table 5 and depicted in Fig. 8. SAR data have the capabilities of in detecting sowing dates of rice precisely^18^.

#### 2017-18

In total, 13 SoS statistics were generated during 2017-18 based on the date of satellite pass and pixels in which the minimum dB occurred viz., 21st August, 2nd September, 14th September, 26th September, 8th October, 20th October, 1^st^ November, 13th November, 25th November, 7th December 2017, 19th December, 31st December 2017 and 12th January 2018. In the study area of Cauvery Delta Zone with a total rice area of 508,581 ha, the largest area of 165,982 ha had the SoS of 1st November followed by 20th October with an area of 63,734 ha. Planting had taken place in an area of 62,296 ha during SoS of 13th November and 59,413 ha during 26th September followed by 45,041 ha during SoS of 8th October. In total, an area of 3.96 lakh ha had the SoS between 26th September to 13th November indicating the major planting period for *samba* season. The early sown area accounted for 38,823 ha from 21st August to 14th September. The late sown area (*Thaladi* and *Navarai*) accounted for 73,292 ha with SoS of 25th November 2017 to 12th January 2018. The SoS from the late sown area indicates the double cropping area, where the delayed harvest of the first crop had resulted in delayed SoS of the second crop.

#### 2018-19

In total, 10 SoS statistics were generated during 2018 based on the date of satellite pass and pixels in which the minimum dB occurred viz., 16th August, 28th August, 9th September, 21st September, 3rd October, 27th October, 8th November, 20th November, 2nd December and 14th December 2018. In the study area of Cauvery Delta Zone with a total rice area of 456,601 ha, the largest area of 110,077 ha had the SoS of 3rd October followed by 21st September with an area of 103,090 ha. Planting took place in an area of 99,165 ha during the SoS of 27th October and 45,637 ha during 9th September followed by 42,573 ha during the SoS of 16th August. In total, an area of 3.77 lakh ha had the SoS between 9th September to 8th November indicating the major planting for *samba* season. The early sown area accounted for 69,310 ha from 16th August to 28th August. The late sown area accounted for 9311 ha with SoS of 20th November to 14th December.

#### 2019-20

A total of 11 SoS statistics were computed i.e., 23rd August, 04th September, 16th September, 28^th^ September, 10th October, 22nd October, 03rd November, 15th November, 27th November, 09th December 2019 and 02nd January 2020. According to derived SoS statistical data, it was noticed that the 22nd October 2019 has the largest area of 130,936 ha, followed by 110,579 ha on the 10th October during s*amba* 2019. Planting was done in an area of 64,760 ha during the SoS of 28th September, 63,730 ha during 15th November and 33,269 ha during the SoS of 03rd November. The SoS between 28th September and 15th November, planting was done in an area of 403,274 ha representing the primary planting time for the *samba* 2019. Early-sown area of 69,795 ha was planted between 23rd August and 16th September. The late sown area accounted for 33,775 ha which was planted between 27th November 2019 and 02nd January 2020.

#### 2020-21

In 2020, 12 SoS statistics were generated based on the date of satellite pass and pixels in which the minimum dB occurred viz., 5th August, 17th August, 29th August, 10th September, 22nd September, 4th October, 16th October, 28th October, 9th November, 21st November, 3rd December and 15th December 2020. In the study area of the Cauvery Delta Zone with a total rice area of 511,714 ha, the largest area of 133,698 ha had the SoS of 16th October followed by 4th October with an area of 101,900 ha. Planting took place in an area of 78,854 ha during SoS of 22nd September and 78,582 ha during 28th October followed by 51,515 ha during SoS of 9th November. In total, an area of 4.44 lakh ha had the SoS between 22nd September and 9th November indicating the major planting period for *samba* season. The early sown area accounted for 49,855 ha from 5th August to 10th September. The late sown area accounted for 17,309 ha with SoS of 21st November, 3rd December to 15th December.

#### 2021-22

A total of 10 SoS statistics were computed i.e., 12th August, 24th August, 05th September, 17th September, 29th September, 11th October, 23rd October, 04th November, 16th November, and 28th November 2021. According to derived SoS statistical data, it was noticed that the 4th November 2021 has the largest area of 103,173 ha, followed by 98,517 ha on 11th October during *samba* season, 2021. Planting was done in an area of 66,230 ha during SoS of 12th August, 83,155 ha during 29th September, and 33,455 ha during SoS of 5th September. The SoS between 29th September and 04th November, planting was done in an area of 345,358 ha representing the primary planting time for the *samba* 2021. Early-sown area of 163,682 ha was planted between 12th August and 17th September. The late sown area accounted for 15,683 ha which was planted between 16th November and 28th November.

#### 2022-23

In total, 10 SoS statistics were generated during 2022-23 based on the date of satellite pass and pixels in which the minimum dB occurred viz., 19th August, 31st August, 12th September, 24th September, 6th October, 30th October, 11th November, 23rd November, 5th December and 17th December 2022. In the study area of the Cauvery Delta Zone with a total rice area of 476,586 ha, the largest area of 133,779 ha had the SoS of 30th October followed by 11th November with an area of 102,240 ha. Planting had taken place in an area of 74,797 ha during SoS of 6th October and 49,237 ha during 24th September followed by 46,199 ha during SoS of 23rd November. In total, an area of 4 lakh ha had the SoS between 24th September to 23rd November indicating the major planting for *samba* season. The early sown area accounted for 42,535 ha from 19th August to 12th September. The late sown area accounted for 27,798 ha with SoS of 5th November to 17th December. Microwave sensors can detect agronomic flooding of rice crops. Sentinel 1 A satellite accurately identifies the rice-growing areas and detects the Start of Season information. SoS in the study area was a function of water release from the canals and was influenced by seasonal variations. SAR data capable for detecting the sowing dates of rice precisely^18^. The SoS from the early sown area indicates the availability of groundwater for utilization. The SoS from the late sown area indicates the double-cropping area, where the delayed harvest of the first crop has resulted in the late SoS of the second crop. The seasonal dynamics of rice crops using Sentinel 1 A and Sentinel 2 A, and the results of the present study were in line with those of previous studies^33^.

**Table 5:** District wise *samba* rice area (ha) corresponding to the start of the season at 12 day intervals in the cauvery Delta region (2017–2023).

2017–18																																												
Districts	21-Aug	02-Sep				14-Sep				26-Sep				08-Oct			20-Oct				01-Nov		13-Nov				25-Nov			07-Dec				19-Dec				31-Dec				12-Jan	Area (ha)	
Thanjavur	0	10,299				4139				20,988				10,667			17,204				25,024		11,573				11,511			9514				5307				0				0	126,226	
Thiruvarur	1023	2363				1422				14,110				9021			19,769				55,370		19,303				2893			5384				1601				0				0	132,258	
Nagappattinam	0	2134				3781				14,480				8913			11,160				52,499		10,644				4827			3875				4913				2186				0	119,411	
Cuddalore	0	0				0				9723				15,918			14,328				30,784		11,927				10,173			2500				2909				383				523	99,170	
Tiruchirapalli	0	12,987				675				112				522			1273				2305		8849				3075			1206				512				0				0	31,516	
Total	1023	27,783				10,017				59,413				45,041			63,734				165,982		62,296				32,479			22,479				15,242				2569				523	508,581	

2018–19																																												
Districts	16-Aug				28-Aug				09-Sep				21-Sep					03-Oct				27-Oct				08-Nov					20-Nov				02-Dec				14-Dec					Area (ha)
Thanjavur	0				7198				21,183				39,151					24,615				27,177				2873					1017				1011				393					124,618
Thiruvarur	15,530				13,129				8512				30,009					36,670				16,823				4605					315				389				37					126,019
Nagapattinam	15,908				5010				12,028				24,423					23,901				19,541				3177					507				576				36					105,107
Cuddalore	10,771				1265				3706				8551					19,964				25,529				4873					755				1054				844					77,312
Tiruchirappalli	364				135				208				956					4927				10,095				4483					1000				1124				253					23,545
Total	42,573				26,737				45,637				103,090					110,077				99,165				20,011					3594				4154				1563					456,601

2019–20																																												
Districts	23-Aug			04-Sep				16-Sep				28-Sep				10-Oct				22-Oct				03-Nov				15-Nov				27-Nov				09-Dec				02-Jan				Area (ha)
Thanjavur	24,118			8806				5822				13,005				27,819				28,792				5771				16,784				7540				2828				0				141,287
Thiruvarur	3807			1748				6145				19,388				26,596				34,899				13,718				15,221				3127				940				0				125,589
Nagapattinam	559			3795				5790				15,507				22,871				40,067				7465				15,482				3593				2631				0				117,761
Cuddalore	0			7				9146				16,853				30,766				22,675				4910				10,513				2822				6151				487				104,331
Tiruchirappalli	12			27				13				6				2528				4503				1404				5730				2780				874				0				17,877
Total	28,496			14,383				26,916				64,760				110,579				130,936				33,269				63,730				19,862				13,425				487				506,844

2020–21																																												
Districts	05-Aug		17-Aug				29-Aug				10-Sep				22-Sep				04-Oct			16-Oct			28-Oct				09-Nov				21-Nov				03-Dec				15-Dec			Area (ha)
Thanjavur	8672		1662				4104				3672				19,608				18,793			36,509			25,409				15,780				3660				1572				1636			141,077
Thiruvarur	2701		594				2811				4198				21,641				21,030			31,604			20,933				19,673				885				1319				363			127,752
Nagapattinam	2127		250				2206				1671				15,582				27,530			28,823			16,112				12,748				679				2823				388			110,938
Cuddalore	785		2140				706				11,471				21,600				25,948			15,213			6425				793				38				2181				700			88,002
Tiruchirapalli	0		0				0				85				422				8599			21,549			9703				2521				90				567				408			43,945
Total	14,285		4646				9827				21,097				78,854				101,900			133,698			78,582				51,515				5352				8462				3495			511,714

2021–22																																												
Districts	12-Aug				24-Aug				5-Sep				17-Sep					29-Sep				11-Oct				23-Oct					4-Nov				16-Nov				28-Nov					Area (ha)
Thanjavur	30,046				15,343				6321				8255					23,554				18,910				10,475					22,144				2276				1847					139,171
Thiruvarur	18,369				8181				13,586				4634					13,790				20,273				6075					40,628				648				844					127,028
Nagapattinam	14,015				7271				2839				8095					25,968				36,058				13,062					13,865				1196				1850					124,219
Cuddalore	0				0				10,709				12,218					19,843				16,817				19,628					19,277				1634				1695					101,821
Tiruchirapalli	3800				0				0				0					0				6460				11,272					7259				2729				964					32,484
Total	66,230				30,795				33,455				33,202					83,155				98,517				60,513					103,173				8483				7200					524,723

2022–23																																												
Districts	19-Aug				31-Aug				12-Sep				24-Sep					06-Oct				30-Oct				11-Nov					23-Nov				05-Dec				17-Dec					Area (ha)
Thanjavur	11,249				633				2400				10,106					19,470				32,495				18,638					14,856				7395				665					117,907
Thiruvarur	5122				810				5035				11,989					17,114				29,657				27,390					8132				5049				216					110,512
Nagapattinam	5339				1083				2391				7815					10,733				26,914				34,912					5622				7402				580					102,791
Cuddalore	0				0				0				19,328					27,481				28,641				15,038					6687				2368				806					100,348
Tiruchirappalli	8473				0				0				0					0				16,073				6263					10,903				3090				227					45,027
Total	30,183				2526				9827				49,237					74,797				133,779				102,240					46,199				25,304				2493					476,586

## Conclusion

Rice area maps and statistics of Cauvery delta districts of Tamil Nadu were generated with an accuracy of 88.5 to 94.5 per cent. The total classified rice area during *samba* season in the Cauvery Delta Zone was 508,581 ha, 456,601 ha, 506,844 ha, 511,714 ha, 524,723 ha and 476,586 ha for the years 2017-18 to 2022-23, respectively. The Start of Season (SoS) maps for *samba* season revealed that the major planting periods for rice were between 26th September to 13th November 2017, 9th September to 8th November 2018, 28th September to 15th November 2019, 22nd September to 9th November 2020, 29th September to 04th November 2021 and 24th September to 23rd November 2022. Over the years, the accuracy of rice area maps generated from Sentinel 1 A SAR data in MAPScape rule-based classifier approach was consistent hence could be recommended for estimating rice area, Start of Season and days of agronomic flooding at regional scale for production forecasting ensuring food security.

## Electronic supplementary material

Below is the link to the electronic supplementary material.

Supplementary Material 1

## Data Availability

All relevant data are included in the manuscript.
