# Supplementary figures and images for: Time series analysis of Sentinel 1 A SAR data to retrieve annual rice area maps and long-term dynamics of start of season
Source: Sci Rep. 2025 Mar 10;15:8202. doi: 10.1038/s41598-025-91655-z (PMC11894211; doi:10.1038/s41598-025-91655-z)

**Year wise area sown data 2017 to 2023**

| **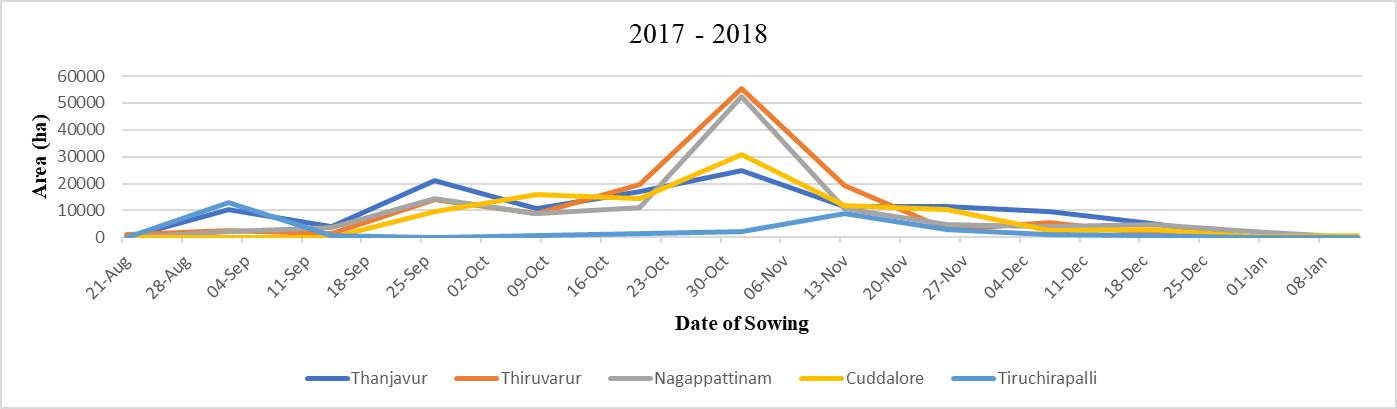** |
| --- |
| **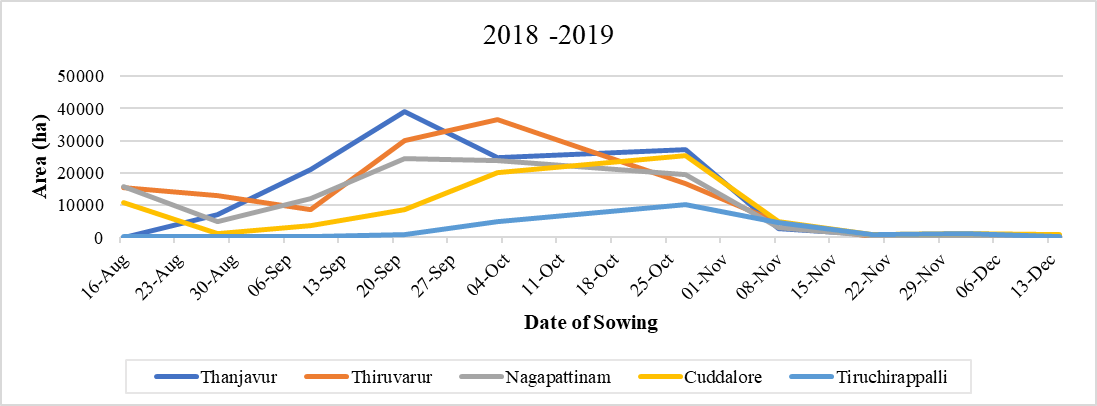** |
| **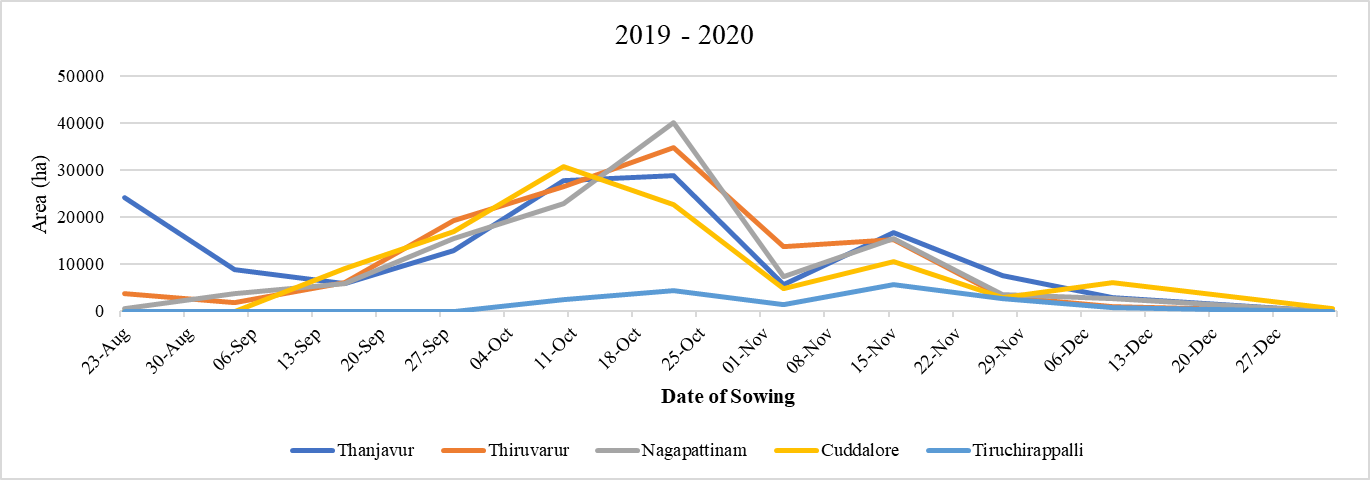** |
| **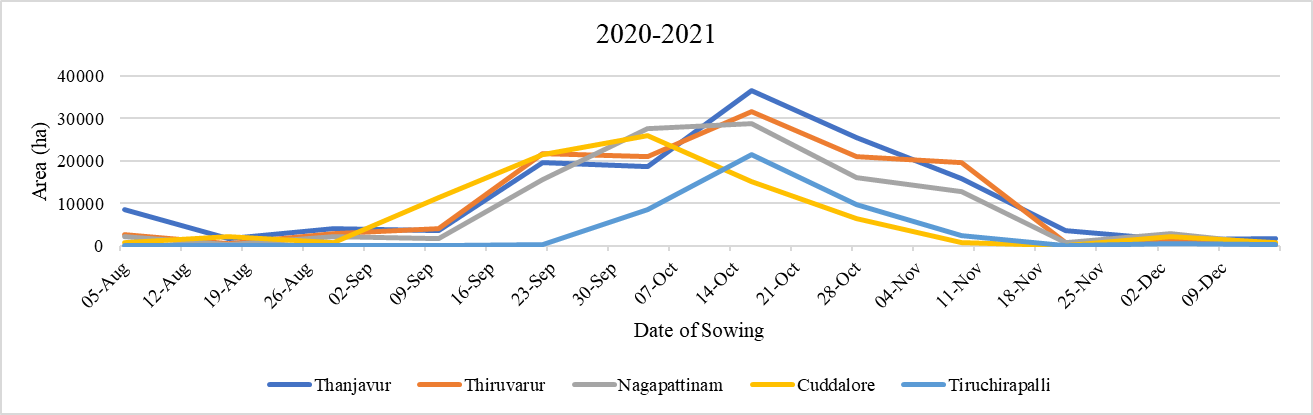** |
| **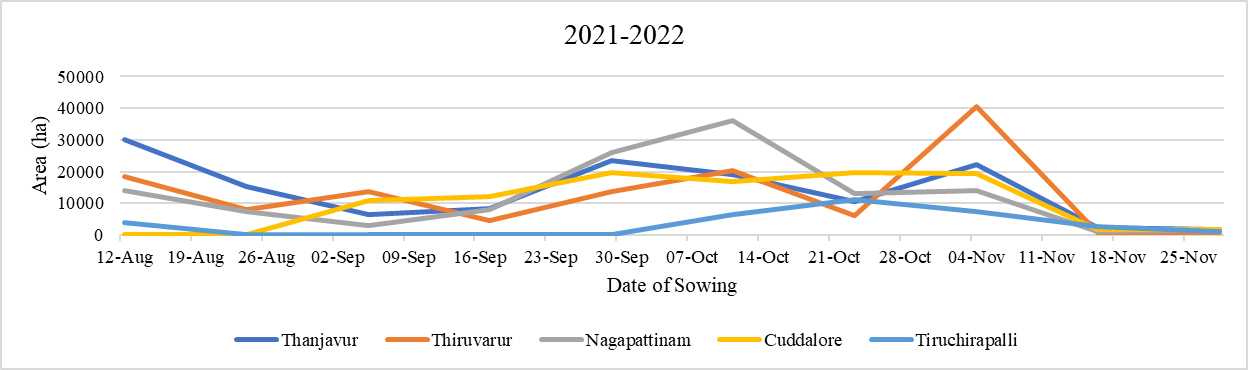** |
| **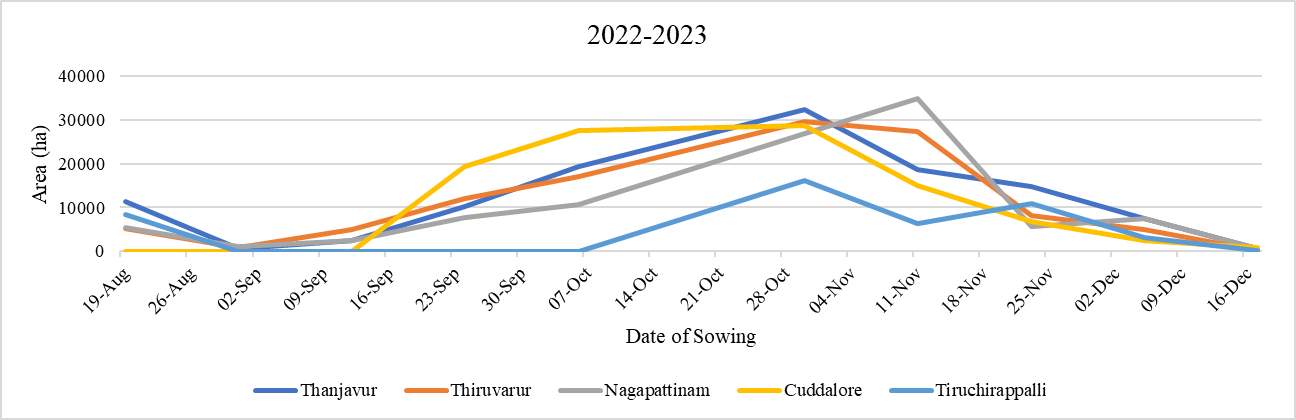** |

Supplement: Supplementary file 1 — Supplementary Material 1 [file 41598_2025_91655_MOESM1_ESM.docx]
